# Untargeted Plasma Metabolomic Profiling in Patients with Major Depressive Disorder Using Ultra-High Performance Liquid Chromatography Coupled with Mass Spectrometry

**DOI:** 10.3390/metabo11070466

**Published:** 2021-07-20

**Authors:** Claudia Homorogan, Diana Nitusca, Virgil Enatescu, Philip Schubart, Corina Moraru, Carmen Socaciu, Catalin Marian

**Affiliations:** 1 Doctoral School, University of Medicine and Pharmacy Victor Babes Timisoara, 300041 Timisoara, Romania; claudia.homorogan@yahoo.com; 2 Department of Biochemistry, University of Medicine and Pharmacy Victor Babes Timisoara, 300041 Timisoara, Romania; nitusca.diana@umft.ro (D.N.); philip.schubart@gmail.com (P.S.); 3 Discipline of Psychiatry, Department of Neurosciences, University of Medicine and Pharmacy Victor Babes Timisoara, 300041 Timisoara, Romania; enatescu.virgil@umft.ro; 4 Eduard Pamfil Psychiatric Clinic, Timisoara County Emergency Clinical Hospital, 300425 Timisoara, Romania; 5 BIODIATECH, Research Center for Applied Biotechnology in Diagnosis and Molecular Therapy, 400478 Cluj-Napoca, Romania; corina_hebristean@yahoo.co.uk (C.M.); csocaciudac@gmail.com (C.S.)

**Keywords:** metabolomics, biomarkers, depression, lipids, LC-MS

## Abstract

Major depressive disorder (MDD) is a neuropsychiatric illness with an increasing incidence and a shortfall of efficient diagnostic tools. Interview-based diagnostic tools and clinical examination often lead to misdiagnosis and inefficient systematic treatment selection. Diagnostic and treatment monitoring biomarkers are warranted for MDD. Thus, the emerging field of metabolomics is a promising tool capable of portraying the metabolic repertoire of biomolecules from biological samples in a minimally invasive fashion. Herein, we report an untargeted metabolomic profiling performed in plasma samples of 11 MDD patients, at baseline (MDD1) and at 12 weeks following antidepressant therapy with escitalopram (MDD2), and in 11 healthy controls (C), using ultra-high performance liquid chromatography coupled with electrospray ionization-quadrupole-time of flight-mass spectrometry (UHPLC-QTOF-(ESI+)-MS). We found two putative metabolites ((phosphatidylserine PS (16:0/16:1) and phosphatidic acid PA (18:1/18:0)) as having statistically significant increased levels in plasma samples of MDD1 patients compared to healthy subjects. ROC analysis revealed an AUC value of 0.876 for PS (16:0/16:1), suggesting a potential diagnostic biomarker role. In addition, PS (18:3/20:4) was significantly decreased in MDD2 group compared to MDD1, with AUC value of 0.785.

## 1. Introduction

Major depressive disorder [MDD] is a recurrent neuropsychiatric disorder with a complex and highly heterogenous etiopathogenesis [1,2]. It is considered a significant health burden since it is associated with high levels of morbidity and mortality pervading all aspects of life [3]. The cognition, mood and behaviour of an individual suffering from MDD can be altered for a total period of 12% years lived with disability, where a major risk factor is represented by suicidal ideation [SI] [4,5]. 

No solitary mechanism can entirely encompass the highly variable nature of this illness, as the emergence of MDD is based upon a set of multifactorial features, acting at genetic, biochemical, neurophysiological, and social levels [6]. 

While twin studies have shown that the heritability of MDD is about 37%, the emerging field of epigenetics revealed that a highly stressful life could lead to depression in some individuals due to a functional polymorphism in the promoter region of the serotonin transporter [5-HTT] [7,8]. In addition, although the pathophysiology and neurobiology of MDD remain to be fully elucidated, it is becoming clear that an altered neural circuitry and the impairment of cellular networks involved in mood and cognition lead to the development of this debilitating condition [9]. MRI techniques have shown that the brains of MDD patients are different in function, structure, and connectivity relative to healthy subjects. Namely, smaller hippocampal volume, diminished communication between subcortical areas mediating negative emotions, and altered neurotransmitter levels illustrate a small part of the current knowledge regarding MDD neurobiology [10,11,12,13].

Interestingly, the neuroendocrine dysregulation that appears in patients suffering from MDD has an important role in understanding its pathophysiology, as external stress alone is involved in regulating an important number of biological pathways [14]. Humoral mediators of immunity, such as cytokines, can have an altered status in MDD patients, explaining mood dysregulation.

Nevertheless, MDD still remains a disorder that lacks an objective and established diagnostic method. Interview-based tools, clinical and behavioral examinations performed by mental health specialists often conduct misdiagnosis, and performing the Structured Clinical Interview for DSM-IV Axis I Disorders [SCID-I] remains disputable due to its moderate reliability [15,16,17]. Furthermore, the lack of accurate and practical biomarkers for early diagnosis in patients developing MDD will affect treatment response and effectiveness. Therefore, the development of reliable diagnostic biomarkers for MDD is warranted and critical for correct and early-onset diagnosis and downstream therapeutic strategies.

In this context, metabolomics is an emerging field that can provide a global snapshot of the metabolic phenotype associated with a particular disorder. In contrast with other “omics” approaches, metabolomics can sample small-molecule metabolites directly, representing the downstream products from the changes that occurred at the genetic, transcriptional, and translational levels [18,19].

Neuropsychiatric disorders, such as MDD, are associated with alterations in metabolic pathways and neurotransmitter concentrations. A broad review report has shown the dysregulated status of amino acids [glutamate, threonine, proline, phenylalanine, arginine, tryptophan, histidine, methionine, glutamine, etc.], neurotransmitters such as GABA, dopamine, serotonin, and metabolites of lipid origin since the brain is an organ with very high lipid content. Metabolomics tools can uncover enormous information regarding the biochemical repertoire of the metabolome and its changes in the biological samples of MDD patients. Therefore, metabolic profiling is considered a crucial tool for novel diagnostic biomarker development and the discovery of the detailed perturbations from the affected biochemical pathways in matters of MDD biology [20].

We performed untargeted metabolomic profiling from plasma samples of MDD patients, before and after treatment with escitalopram, and from plasma samples of healthy volunteers, by ultra-high performance liquid chromatography coupled with electrospray ionization-quadrupole-time of flight-mass spectrometry [UHPLC-QTOF-[ESI+]-MS]. Our aim was (1) to investigate possible differences in metabolites levels between patients before treatment and healthy volunteers to assess their potential diagnostic biomarker role for MDD detection, and (2) to analyse the antidepressant effect of escitalopram by comparing the metabolome of the same patients at baseline, and after a 12-week follow-up period, with escitalopram antidepressant treatment. 

## 2. Results

### 2.1. Metabolites identiFication 

The Human Metabolomic Database (HMDB) was used for metabolites identification with putative names. After sequential filtration of small signals/noises (<5) and peak intensities (<1000), a number of common 61 metabolites were found, as presented in Table 1, with their corresponding m/z values. 

Mean, SD, SD/mean values, and the ratios between tested groups (C—healthy controls, MDD1—patients before treatment, MDD2—patients after treatment) were calculated to analyse the differences in metabolite levels between groups. In general, metabolites levels of lipid origin were increased after treatment when compared to patients before treatment and to controls. 

In addition, when comparing patient groups before and after treatment, we observed a general increase in signal intensity and therefore in metabolites percentages. We observed an increase in signal intensity of over 25% (for leukotriene C4, 20:1 cholesterol ester, adenosine monophosphate, PG, and PC, data not shown). 

### 2.2. Metabolomic Profiles of Controls and Patients before Treatment (C vs. MDD1)

**Figure 1:**
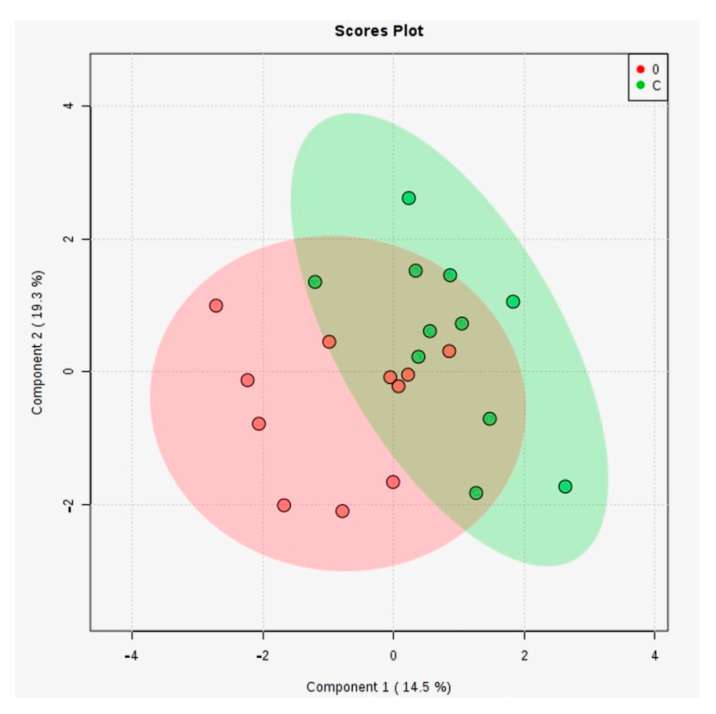
2D score plots of PLS-DA analysis for control groups (C) vs. MDD1 (code 0).

First, we performed a multivariate statistical analysis, namely Principal Component Analysis (PCA, Supplementary Figure S1) and Partial Least Square Discriminant Analysis (PLS-DA, Figure 1) for groups C vs. 0 (MDD1). 

While the PCA plot does not show a significant separation of groups (see Supplementary Figure S2), the PLSDA graph reflects a better discrimination. In this case, the covariance was 33.8% for the first two components. The cross-validation algorithm showed an accuracy of 0.5, R2 = 0.8 and non-significant Q2 values (data not shown), the model having low predictability.

The m/z values with the VIP (Variable Importance in Projection) value > 0.8 were selected (as mentioned in Table 2), which should be taken into consideration. In addition, the names of these compounds were identified through Human Metabolome Database (HMDB), as well as the *p*- and fold change (FC) values.

Considering a threshold of *p* ˂ 0.005, two metabolites (PS (16:0/16:1) and PA (18:1/18:0)) were shown to be statistically significant. Both increased in then MDD1 samples when compared to healthy subjects. In this case, FC and *p* for PS (16:0/16:1) were 1.699 and *p* ˂ 0.001, respectively. For PA (18:1/18:0), these values were 2.508 and 0.005, respectively (Table 2). 

**Figure 2:**
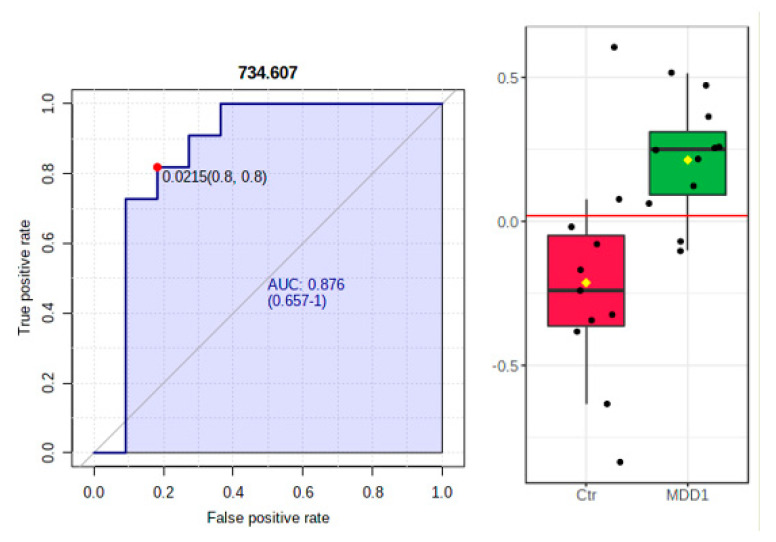
ROC analysis based on area under ROC curve (AUROC) >0.8 for PS (16:0/16:1) (*m/z* = 734.607). The mean values are higher in the MDD1group (before treatment).

The results of the ROC analysis are summarized in Table 3 and shown in Figure 2 (left and right). 

### 2.3. Metabolomic Profiles of Patients, before and after Treatment (MDD1 vs. MDD2) 

**Figure 3:**
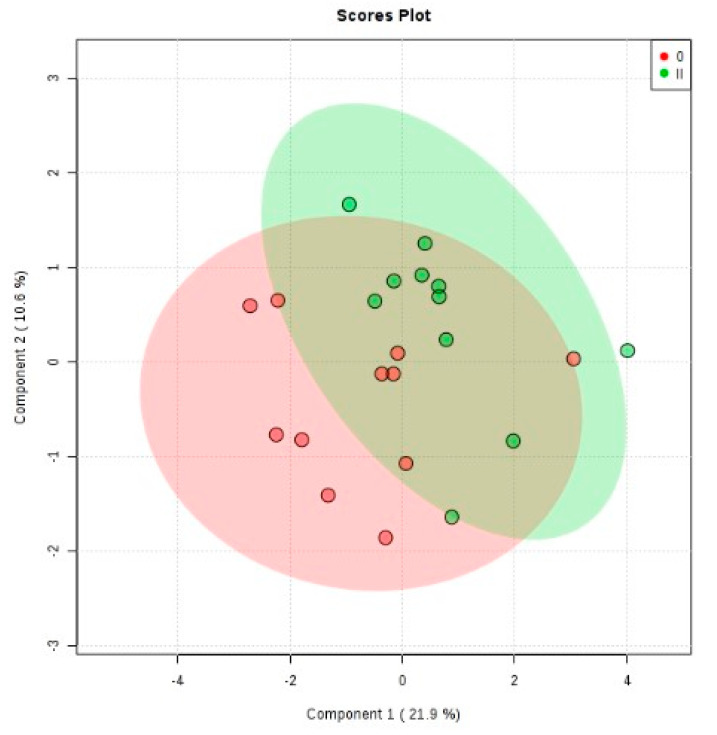
2D score plots for PLS-DA analysis for groups MDD2 (code II) vs. MDD1 (Code 0).

Multivariate statistical analyses (PCA, PLS-DA, m/z values with VIP > 0.2 on all five components) were performed and are presented in Figure 3 and Table 4, respectively.

Also, in this case, while the PCA plot does not show a significant separation of groups (see Supplementary Material), the PLSDA graph reflects a better discrimination. In this case, the covariance was 31.5% for the first two components. The cross-validation algorithm showed an accuracy of 0.4, R2 = 0.8 and non-significant Q2 values (data not shown), the model having low predictability.

Table 4 mentions the identification of molecules and the VIP, *p*- and fold change (FC) values.

**Figure 4:**
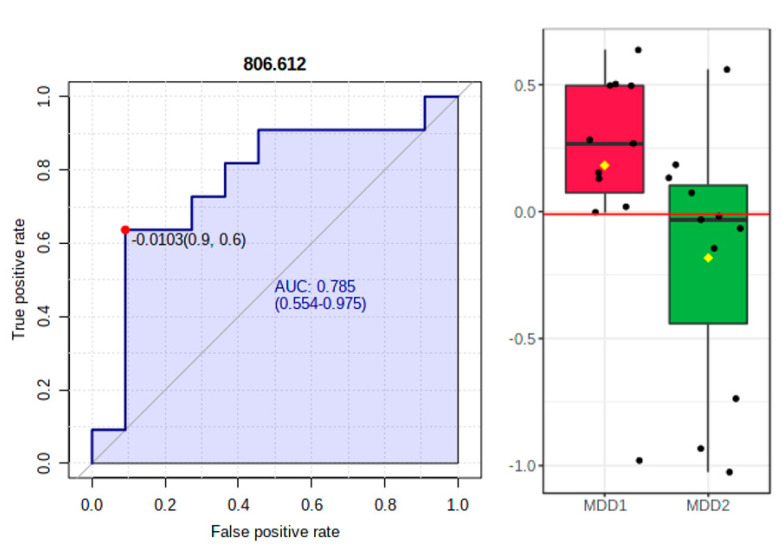
ROC analysis based on area under ROC curve (AUROC)> for PS (*m/z* = 806.612).

The *t*-test for univariate statistical analysis showed only PS (18:3/20:4) as statistically significant (*p* = 0.028) with FC of 0.720. The ROC analysis displayed a moderately high diagnostic value for PS (18:3/20:4), with an AUC value of 0.785 (Figure 4, left and right). Table 5 presents the identification of molecules with AUC values above 0.650, which may be considered putative biomarkers of differentiation between MDD1 and MDD2 groups (before and after treatment).

## 3. Discussion 

The present study found significant differences in two metabolites levels [[PS [16:0/16:1] and PA [18:1/18:0]] in MDD patients before antidepressant treatment relative to healthy controls. Plasma levels of PS [16:0/16:1] and PA [18:1/18:0] were significantly higher [*p* < 0.05] in the patients group at baseline. Moreover, ROC analysis based on AUC values revealed a high diagnostic value for PS [16:0/16:1] [AUC value of 0.876]. On the other hand, the PS [18:3/20:4] levels were significantly decreased in patients after treatment with escitalopram compared to the same patients at baseline, and ROC analysis for PS [18:3/20:4] displayed a moderately high diagnostic value for this metabolite [AUC of 0.785].

To our knowledge, our study confirmed and corroborated previous literature findings regarding plasma levels of PS in MDD patients. Meta-analysis studies have undertaken mounting efforts to integrate particular metabolic alterations found in the biological samples of MDD patients for more facile biomarker development and elucidation of the complex molecular mechanisms that make up the heterogenous neurobiology of MDD. In this context, one study found that the fatty acid biosynthesis was found among the top-ranked altered metabolic pathways in matters of MDD development and that MDD patients had significantly higher levels of asymmetric dimethylarginine, tyramine, 2-hydroxybutyric acid, phosphatidylcholine [32:1], and taurochenodesoxycholic acid [21]. Moreover, Knowles et al. (2017) found while analysing the lipidome in MDD, that the ether-phosphatidylcholines were the lipids that presented the highest pleiotropy, PC having the largest endophenotype ranking value [ERV] of 0.13 [22]. 

Yet another phospholipidomic profiling study performed on a mouse model of depression induced by chronic unpredictable stress [CUS] revealed a significantly higher level of phospholipids in the brain and myocardium of mice after unpredictable chronic stress conditions. Thus, it is becoming increasingly clear that depression has an important impact on the brain lipidome, and analysing these small molecule metabolites could provide insight into the lipid metabolism disorder in MDD and grant promising biomarker potential for the diagnostic of this debilitating illness [23]. 

Furthermore, Liu et al. (2016) attempted to analyse the relationships of specific metabolites with depression severity in a plasma lipidomic report. Among numerous metabolites studied, the levels of phospholipids were significantly increased in MDD patients and had highly positive relationships with depression severity measured by the Hamilton Depression Scale [HAMD]. Furthermore, the same report found excellent diagnostic value in moderate [M] and severe [S] MDD for a combinational lipid panel including LPE 20:4, PC 34:1, PI 40:4, SM 39:1, 2, and TG 44:2. Sensitivity, specificity and AUC values for the discrimination between MDD and healthy controls were 75.6%, 92.3% and 0.855, respectively for M-MDD and 80.0%, 92.3% and 0.931, respectively for S-MDD [24].

Interestingly, another lipid metabolite we identified as being significantly altered in plasma samples of MDD patients relative to healthy volunteers was phosphatidic acid [PA [18:1/18:0]]. To our knowledge, there was no previously published report that assessed the diagnostic value of PA [18:1/18:0] or PS in relationship with MDD diagnosis. Nevertheless, despite having an inferior diagnostic performance compared to PS, PA [18:1/18:0] is part of the altered lipidomic pattern, which is specific to psychiatric illnesses [25]. Moreover, our study revealed that the levels of PA [18:1/18:0] remain elevated after antidepressant treatment with escitalopram, suggesting that these metabolites are involved in MDD pathophysiology in a rather direct fashion, independent from medication, leastways with escitalopram. Other reports have identified and elucidated the structure of escitalopram-specific metabolites. At the same time, 1H NMR studies revealed that lipid metabolism-related metabolites are generally altered in patients treated with lithium and may be linked to this particular medication [26,27,28].

Our results revealed changes in metabolites level of lipid and phospholipid origin, containing linoleic/linolenic and arachidonic acid, PS and PC, and an increase, although not significant, of PC [18:2 and 18:1] and lysoderivatives [LPC 18:2] after treatment. Metabolic pathways of membrane phospholipids also seem to be involved in the metabolic alterations that occur in MDD, which might be related to antidepressant treatment. In addition, superior unsaturated acids such as dihomo-γ-linolenic acid and arachidonic acid have been found to be involved in MDD development, also in patients receiving antidepressant treatment [escitalopram] compared to the same patients at baseline.

Taken together, our data followed previously published reports and revealed once again that altered metabolites in MDD are, to a certain extent, of lipid origin. Glycerophospholipids are mainly involved in membrane formation and trafficking, and alterations at these levels could dramatically influence the global lipidome of MDD, since lipids are organized hierarchically and are strongly interconnected. One hypothesis is that when phospholipase A2 [PLA2] converts PC into arachidonic acid [AA], inflammatory molecules such as prostaglandins are rapidly produced, leading to a neuro-inflammation process, well-documented in MDD development [29,30,31,32]. It is known that AA alone can modulate membrane polarization and fluidity, thus being directly involved in neural cell function and MDD symptomatology [22,33]. Furthermore, PA and its metabolite [diacylglycerol], together with other lysophosphatidylcholines, can also cause membrane bending and destabilization, potentially contributing to the development of MDD [25].

Furthermore, we found in our study that PS [18:3/20:4] levels significantly dropped after administration of SSRI antidepressant treatment [escitalopram]. While it is difficult to draw confident conclusions regarding the antidepressant effect upon the phospholipidomic profile of MDD patients, it is clear that different SSRI medication expresses different effects on the lipid profile. A handful of studies have demonstrated that the administration of escitalopram significantly alters cholesterol levels, acylcarnitines, phosphatidylcholine, s and etherphospholipids, with the exact pharmacological mechanism remaining elucidated [34,35].

Nevertheless, our study had some limitations that need to be acknowledged, which arise primarily from the limited sample size. Validation of PS and PA in independent cohorts is another crucial step to specifically analyse its potential biomarker role, diagnostic value and correlation with escitalopram. Thus, future large-scale powered studies need to be conducted to definitively attribute the biomarker potential of PS and PA in MDD diagnosis and their role in the development of this debilitating illness.

## 4. Materials and Methods

### 4.1. Study Population and Specimen Collection

A total number of 22 participants were enrolled in this study, out of which 11 were patients suffering from MDD, and 11 were healthy subjects. The controls (C) were matched by age and gender (between 18–65 years) with the patients. Diagnostic and sampling methods were performed as previously described [36]. Briefly, all patients included in the study fulfilled the DSM-IV-TR diagnostic criteria for MDD. This was a follow-up research study with two time points (baseline and 12 weeks after administration of a 10 mg daily dose of escitalopram). Inclusion and exclusion criteria were the same as previously reported [36].

Clinical and demographic characteristics of the study population are presented in Table 6.

Before specimen collection, all subjects provided informed consent for the use of their biological samples, and the study was approved by the Ethical Committee of the participating institutions, which is in accordance with the 1964 Declaration of Helsinki and its later amendments. 

Blood samples (5 mL) were collected in the morning from MDD patients at baseline (which we further denominated group MDD1, code 0) and 12 weeks after administration of antidepressant medication (escitalopram, group MDD2, code II) in K3EDTA-coated tubes. Healthy controls (group C) had their blood samples collected only once at baseline. Plasma was separated via centrifugation within 1 h after collection, aliquoted and stored at −80 °C for one week. Before use, samples were centrifuged for 5 min at 15,000× *g* (4 °C). The thawing was performed under sonication and vortex (5 min) before the LC-MS analysis, at 30 min before refreezing.

### 4.2. Metabolites Extraction from Plasma 

A volume of 0.6 mL methanol (99%) was added for each volume of 0.2 mL of plasma, and the mixture was vortexed to precipitate proteins for 30 seconds. The mixture was then kept for 5 min in an ultrasonication instrument, followed by 5 min at −20 °C. The supernatant was collected after centrifugation at 10,000 rpm for 10 min (4 °C). For each volume of 0.5 mL supernatant, we added 0.5 ml double-distilled water and 0.5 mL analytical grade chloroform. The mixture was once again vortexed, and centrifuged at 10,000 rpm for 10 min. 0.5 mL of the superior phase (the methanol-water extract) was filtered through 0.2 µm nylon filters and subsequently injected in the HPLC column. The standard procedure (SOP) we used considers the elimination of plasma triglycerides by chloroform extraction.

### 4.3. UHPLC-QTOF-(ESI+)-MS Analysis

Plasma metabolomic profiling was performed using ultra-high-performance liquid chromatography coupled with electrospray ionization-quadrupole-time of flight-mass spectrometry (UHPLC-QTOF-(ESI+)-MS) in a ThermoFisher Scientific UHPLC Ultimate 3000 instrument equipped with a quaternary pump, Dionex delivery system, and MS detection equipment with MaXis Impact (2012 version, Bruker Daltonics).

A 3-µm Intrada (50 × 3 mm) column was used (Imtakt, Kyoto, Japan). The stationary phase has a 3-µm particle size, and pore size of 30 nm (300 Å). The mobile phase consisted of solution A (water + 0.1% formic acid) and solution B (acetonitrile + 0.1% formic acid). The gradient was: 5 to 15% A (0–3 min), 15–50% A (3–6 min), 50–95% (6–9 min), isocratic until 15 min, and afterward decreased from 95 to 5% (15–20 min). The elution time was set for 20 min. The volume of injected extract was 5 µl, the column temperature at 40 °C, and the flow was set at 0.5 mL/min.

Metabolites identification was performed using a mass spectrometer, using the following parameters: the pressure for the nebulizing gas was set at 2.8 bar, the flux and temperature of the dry gas were set at 12 L/min, and 300 °C, respectively. Before each injection step, we added a calibration solution (sodium formate) in the UHPLC-QTOF-(ESI+)-MS. TofControl 3.2, HyStar 3.2, Data Analysis 4.1 and Profile Analysis 2.1 (Bruker Daltonics) software were used for instrument control and data processing. The standard deviation of compounds from the calibration solution was less than 1 ppm, and the capillary voltage was 4500 V.

### 4.4. Data Processing and Statistical Analysis

The Base Peak chromatograms and all MS spectra were firstly processed by Compass DataAnalysis 4.2 (Bruker Daltonics, GmbH, Bremen, Germany) using Find Molecular Feature (FMF) algorithm.

Profile Analysis 2.1 (Bruker Daltonics, GmbH, Bremen, Germany) was further used for matrix generation from the obtained FMF. Parameters such as time alignment, spectral background extraction, MS recalibration, normalization by the sum of the bucket values in analysis and an 80% bucket filter was used. The blank *m/z* values were subtracted from the samples variable.

Next, the MetaboAnalyst v5.0 online software was used for univariate and multivariate analysis. For multivariate analysis, KNN missing value estimation, none data filtering, normalization by sum, log data transformation and Pareto data scaling was used. For univariate analysis, no missing value estimation, data filtering, normalization by sum and non-parametric analysis were used to further model the data. 

The multivariate analysis consisted of the representation of Fold Change, Volcano Plot, Principal Component Analysis (PCA), Partial Least Squares Discriminant Analysis (PLSDA), Random Forest, finding correlations between the samples and variables (*m/z* values) as a Heatmap. Finally, using the Biomarker Analysis, the Receiver Operating Curves (ROC) were obtained, and the values of areas under ROC curves (AUC) were calculated, the molecules being ranked according to their sensitivity/specificity.

The identification of molecules that can be considered potential biomarkers was made using the two most relevant databases, LIPID MAPS^®^ Lipidomics Gateway (https://www.lipidmaps.org/data/structure/LMSDSearch.php, accessed on 15 May 2021) and Human Metabolome Database (https://hmdb.ca/, accessed on 15 May 2021).

## 5. Conclusions

MDD is a severe neuropsychiatric illness that presently lacks accurate, reliable and early diagnostic tools. The powerful field of metabolomics holds great promise for the discovery and development of novel circulating biomarkers for this disease since it is capable of portraying the complex metabolomic changes that occur at the levels of small molecule metabolites in MDD patients relative to healthy controls. We reported an untargeted metabolomics profiling in plasma samples of MDD patients before and after treatment with escitalopram and healthy controls and found significantly increased levels of phosphatidylserine (PS (16:0/16:1)) and phosphatidic acid (PA (18:1/18:0)), and decreased levels of PS (18:3/20:4) after antidepressant treatment. 

Future validation studies are warranted to confirm our findings. Integrating all the altered metabolome data could potentially aid in the development of minimally invasive panel biomarkers that could represent optimized diagnostic tools and enrich current knowledge regarding MDD pathophysiology and antidepressant effects on the lipid profile. In addition, by uncovering the metabolic alterations in MDD patient samples, one can gain more insight into the pathophysiology and neurobiology of this complex and debilitating disorder for more facile disease management and therapeutic strategy selection.

## Figures and Tables

**Table 1:** The m/z values and putative identification of metabolites separated by UHPLC-ESI+MS.

No.	*m/z* [M+H]+	Metabolites Identification
1	256.275	Palmitamide
2	301.154	Andrenosterone
3	304.274	Arachidonyl alanine, Palmitoyl ethanolamine
4	313.288	Eicosanoic (arachidic) acid
5	318.255	Leucyl-Tryptophan
6	331.296	Deoxycorticosterone
7	339.316	11,12-DiHETrE
8	341.321	9-Hexadecenoylcholine
9	347.141	Corticosterone
10	348.326	Adenosine monophosphate
11	353.282	Prostaglandin E2/D2
12	357.327	Prostaglandin F1a
13	359.33	Tetracosapentaenoic acid
14	369.368	Thromboxane B3
15	381.315	Sphinganine -1 Phosphate
16	467.405	Cholesterol sulfate
17	483.402	11-beta-Hydroxyandrosterone-3-glucuronide
18	496.365	LPC (16:0)
19	518.347	LPC (18:3)
20	520.366	LPC (18:2)
21	522.381	LPC (18:1)
22	524.397	LPC (18:0)
23	542.349	LPC (20:5)
24	544.366	LPC (20:4)
25	563.578	DG( 18:2/14:1/0:0)
26	609.549	DG (17:1/18:0/0:0) [iso2]
27	623.529	Ceramide (d18:1/22:0)
28	625.545	Ceramide (d18:0/22:0)
29	626.48	Leukotriene C4
30	639.526	PA(32:5)
31	641.541	DG (18:1/20:5/0:0)
32	663.488	PG (14:1/14:1)
33	677.021	SM (d18:0/14:0)
34	679.019	Cer (d18:1/26:0)
35	679.547	20:1 Cholesterol ester
36	701.529	PA (18:1/18:1)
37	703.611	PA (18:1/18:0)
38	712.521	PE (16:0/18:4)
39	732.591	SM (d18:1/18:0)
40	734.504	SM (d18:0/18:0)
41	734.607	PS (16:0/16:1)
42	739.57	PA (21:0/18:4)
43	756.593	PS (16:1/18:3)
44	758.61	PS (16:1/18:2)
45	760.624	PS (16:1/18:1)
46	761.553	TG (13:0/16:1/16:1) [iso3]
47	768.629	PC (18:2/17:2)
48	772.624	PG (18:1/18:3)
49	780.593	PC (18:3/18:2)
50	782.611	PC (18:2/18:2)
51	784.625	PC (18:1/18:2)
52	786.642	PC (18:1/18:1)
53	792.636	PS (18:0/18:0)
54	806.612	PS (18:3/20:4)
55	808.626	PS (18:2/20:4)
56	810.643	PS (18:1/20:4)
57	814.62	PS (18:1/20:4)
58	825.61	TG (16:0/16:0/18:2 (9Z,12Z)) [iso3]
59	834.644	PC (18:0/22:6)
60	905.726	TG (16:0/18:0/21:0) [iso6]
61	927.71	TG (18:1/19:0/20:2) [iso6]

Abbreviations: LPC = lysophosphatidylcholine; DG = diacylglycerol; PA = phosphatidic acid; PG = phosphoglycerol; SM = sphingomyelin; PE = phosphatidylethanolamine; PS = phosphoserine; TG = triacylglycerol; PC = phosphatidylcholine.

**Table 2:** Metabolites with VIP > 0.8 on all components in group C vs. 0 (MDD1), *p*- and FC values.

No.	Metabolite Identification [M+H]+	RT (min)	*m/z*	VIP	*p*-value	FC	FDR
1	PS (16:0/16:1)	10.5	734.607	2.813	<0.001	1.699	0.009
2	PS (18:1/20:4)	10.39	810.643	1.887	0.133	1.283	0.888
3	PC (18:2/18:2)	10.24	782.611	1.884	0.065	1.272	0.717
4	PA (18:1/18:0)	8.93	703.611	1.859	0.005	2.508	0.086
5	Deoxycorticosterone	8.11	331.296	1.406	0.401	0.826	0.888
6	LPC (18:2)	9.33	520.366	1.358	0.217	0.842	0.888
7	PG (18:1/18:3)	10.37	772.624	1.308	0.332	1.218	0.888
8	LPC (20:4)	9.37	544.366	1.158	0.401	1.231	0.888

PS = phosphatidylserine; PC = phosphatidylcholine; PA = phosphatidic acid; LPC = lysophosphatidylcholine; PG = phosphatidylglycerol; FDR = false discovery rate.

**Table 3:** The AUC values above 0.650 and the identification of molecules to be considered putative biomarkers differentiation between controls and MDD1 group (before treatment).

Molecule	AUC
PS (16:0/16:1)	0.876
PA (18:1/18:0)	0.777
PS (18:1/20:4)	0.678

**Table 4:** Metabolites levels with VIP > 0.2 on all components in comparison group MDD2 vs. MDD1 *p*- and FC values.

No.	Metabolite Identification [M+H]+	RT (min)	*m/z*	VIP	*p*-Value	FC	FDR
1	PS(18:3/20:4)	10.12	806.612	2.376	0.028	0.720	0.926
2	Deoxycorticosterone	8.11	331.296	1.782	0.365	1.272	0.964
3	PC (18:1/18:1)	10.45	786.642	1.761	0.171	1.174	0.964
4	PC (18:2/17:2)	10.85	768.629	1.656	1.000	1.011	0.964
5	PG (18:1/18:3)/ PG (18:2/18:2)	10.37	772.624	1.573	0.438	0.804	0.964
6	Sphinganine 1-phosphate	8.27	381.315	1.546	0.171	1.169	0.964
7	PC (18:1/18:3)/ PC (18:2/18:2)	10.24	782.611	1.431	0.193	0.853	0.964
8	LPC (20:4)	9.37	544.366	1.232	0.748	0.824	0.964
9	Adenosine monophosphate	8.10	348.326	1.063			0.964
10	LPC (20:5)	9.33	542.349	0.988	0.438	0.847	0.964
11	LPC (18:2)	9.33	520.366	0.919			0.964

PS = phosphatidylserine; PC = phosphatidylcholine; PA = phosphatidic acid; LPC = lysophosphatidylcholine; PG = phosphatidylglycerol.

**Table 5:** The AUC values above 0.650 and the identification of molecules to be considered putative biomarkers of differentiation between MDD1 and MDD2 group.

Name	AUC	Changes MDD2 vs. MDD1
PS (18:3/20:4)	0.785	Decrease
Sphinganine 1-phosphate	0.678	Decrease
PC (18:1/18:1)	0.678	Increase
PC (18:1/18:3) or PC (18:2/18:2)	0.669	Decrease
PC (18:1/18:2)	0.669	Increase
PS (16:1/18:1)	0.669	Decrease

**Table 6:** Characteristics of MDD patients and controls enrolled in the study.

Variables	MDD Patients (*n* = 11)	Healthy Controls (*n* = 11)
Age in years (median)	43.81	45
Gender		
Male	3	3
Female	8	8
HDRS-17 (median score)		
Before treatment	23.27	
After treatment	5.81	

## Data Availability

All data is published together with the manuscript. Data is available from corresponding author, upon request.

## Supplementary Materials

The following are available online at https://www.mdpi.com/article/10.3390/metabo11070466/s1, Figure S1: PCA (2D) in control groups vs patients before treatment (C vs. MDD1); Figure S2: PCA (2D) in patients before and after treatment (MDD1 vs MDD2)

## References

[1] Möller H.J., Seemüller F.H., Riedel M. (2009). Time course of response and remission during antidepressant treatment. Medicographia.

[2] Chiriţă A.L., Gheorman V., Bondari D., Rogoveanu I. (2015). Current understanding of the neurobiology of major depressive disorder. Rom. J. Morphol. Embryol..

[3] Hays R.D., Wells K.B., Sherbourne C.D., Rogers W., Spritzer K. (1995). Functioning and well-being outcomes of patients with depression compared with chronic general medical illnesses. Arc. Gen. Psych..

[4] Mitchell A.J., Vaze A., Rao S. (2009). Clinical diagnosis of depression in primary care: A meta-analysis. Lancet.

[5] Setoyama D., Kato T.A., Hashimoto R., Kunugi H., Hattori K., Hayakawa K., Kanba S. (2016). Plasma Metabolites Predict Severity of Depression and Suicidal Ideation in Psychiatric Patients-A Multicenter Pilot Analysis. PLoS ONE.

[6] Belmaker R.H., Agam G. (2000). Major depressive disorder. N. Engl. J. Med..

[7] Sullivan P.F., Neale M.C., Kendler K.S. (2000). Genetic epidemiology of major depression: Reviews and metaanalysis. Am. J. Psychiatry.

[8] Caspi A., Sugden K., Moffitt T.E., Taylor A., Craig I.W., Harrington H., Poulton R. (2003). Influence of life stress on depression: Moderation by a polymorphism in the 5-HTT gene. Science.

[9] Roy B., Dunbar M., Shelton R.C., Dwivedi Y. (2017). Identification of MicroRNA-124-3p as a Putative Epigenetic Signature of Major Depressive Disorder. Neuropsychopharmacology.

[10] Videbech P., Ravnkilde B. (2004). Hippocampal volume and depression: A meta-analysis of MRI studies. Am. J. Psychiatry.

[11] Sheline Y.I., Gado M.H., Kraemer H.C. (2003). Untreated depression and hippocampal volume loss. Am. J. Psychiatry.

[12] Anand A., Li Y., Wang Y., Wu J., Gao S., Bukhari L., Lowe M.J. (2003). Activity and connectivity of brain mood regulating circuit in depression: A functional magnetic resonance study. Biol. Psychiatry.

[13] Sen S., Duman R., Sanacora G. (2008). Serum brain-derived neurotrophic factor, depression, and antidepressant medications: Meta-analyses and implications. Biol. Psychiatry.

[14] Raison C.L., Miller A.H. (2003). When not enough is too much: The role of insufficient glucocorticoid signaling in the pathophysiology of stress-related disorders. Am. J. Psychiatry.

[15] Zajecka J.M. (2003). Treating depression to remission. J. Clin. Psychiatry.

[16] Schmidt H.D., Shelton R.C., Duman R.S. (2011). Functional biomarkers of depression: Diagnosis, treatment, and pathophysiology. Neuropsychopharmacology.

[17] First M.B., Gibbon M., Hilsenroth M.J., Segal D.L. (2004). The Structured Clinical Interview for DSM-IV Axis I Disorders (SCID-I) and the Structured Clinical Interview for DSM-IV Axis II Disorders (SCID-II). Comprehensive Handbook of Psychological Assessment.

[18] German J.B., Hammock B.D., Watkins S.M. (2005). Metabolomics: Building on a century of biochemistry to guide human health. Metabolomics.

[19] Dettmer K., Aronov P.A., Hammock B.D. (2007). Mass spectrometry-based metabolomics. Mass. Spectrom. Rev..

[20] Quinones M.P., Kaddurah-Daouk R. (2009). Metabolomics tools for identifying biomarkers for neuropsychiatric diseases. Neurobiol. Dis..

[21] Pu J., Liu Y., Zhang H., Tian L., Gui S., Yu Y., Xie P. (2020). An integrated meta-analysis of peripheral blood metabolites and biological functions in major depressive disorder. Mol. Psychiatry.

[22] Knowles E.E., Huynh K., Meikle P.J., Göring H.H.H., Olvera R.L., Mathias S.R., Glahn D.C. (2017). The lipidome in major depressive disorder: Shared genetic influence for ether-phosphatidylcholines, a plasma-based phenotype related to inflammation, and disease risk. Eur. Psychiatry.

[23] Faria R., Santana M.M., Aveleira C.A., Simões C., Maciel E., Melo T., Domingues M.R.M. (2014). Alterations in phospholipidomic profile in the brain of mouse model of depression induced by chronic unpredictable stress. Neuroscience.

[24] Liu X., Li J., Zheng P., Zhao X., Zhou C., Hu C., Xu G. (2016). Plasma lipidomics reveals potential lipid markers of major depressive disorder. Anal. Bioanal. Chem..

[25] Walther A., Cannistraci C.V., Simons K., Durán C., Gerl M.J., Wehrli S., Kirschbaum C. (2018). Lipidomics in Major Depressive Disorder. Front. Psychiatry.

[26] Bandu R., Lee H.J., Lee H.M., Ha T.H., Lee H.J., Kim S.J., Kim K.P. (2018). Liquid chromatography/mass spectrometry-based plasma metabolic profiling study of escitalopram in subjects with major depressive disorder. J. Mass. Spectrom..

[27] Hashimoto K. (2018). Metabolomics of Major Depressive Disorder and Bipolar Disorder: Overview and Future Perspective. Adv. Clin. Chem..

[28] Sussulini A., Prando A., Maretto D.A., Poppi R.J., Tasic L., Banzato C.E.M., Arruda M.A.Z. (2009). Metabolic profiling of human blood serum from treated patients with bipolar disorder employing 1H NMR spectroscopy and chemometrics. Anal. Chem..

[29] Van Meer G., Voelker D.R., Feigenson G.W. (2008). Membrane lipids: Where they are and how they behave. Nat. Rev. Mol. Cell. Biol..

[30] Quehenberger O., Dennis E.A. (2011). The human plasma lipidome. N. Engl. J. Med..

[31] Sud M., Fahy E., Cotter D., Brown A., Dennis E.A., Glass C.K., Subramaniam S. (2007). LMSD: LIPID MAPS structure database. Nucleic. Acids. Res..

[32] Mondelli V., Vernon A.C., Turkheimer F., Dazzan P., Pariante C.M. (2017). Brain microglia in psychiatric disorders. Lancet Psychiatry.

[33] Demirkan A., Isaacs A., Ugocsai P., Liebisch G., Struchalin M., Rudan I., van Duijn C.M. (2013). Plasma phosphatidylcholine and sphingomyelin concentrations are associated with depression and anxiety symptoms in a Dutch family-based lipidomics study. Ays. J. Psychiatr. Res..

[34] Olguner Eker Ö., Özsoy S., Eker B., Doğan H. (2017). Metabolic Effects of Antidepressant Treatment. Noro. Psikiyatr. Ars..

[35] Mahmoudian Dehkordi S., Ahmed A.T., Bhattacharyya S., Han X., Baillie R.A., Arnold M., Kaddurah-Daouk R. (2020). Alterations in Acylcarnitines, amines, and lipids inform about mechanism of action of citalopram/escitalopram in major depression. bioRxiv.

[36] Enatescu V.R., Papava I., Enatescu I., Antonescu M., Anghel A., Seclaman E., Marian C. (2016). Circulating Plasma Miro RNAs in Patients with Major Deppresive Disorder Treated with Antidepressants: A Pilot Study. Psychiatry Investig..

