# Supplementary figures and images for: Untargeted Plasma Metabolomic Profiling in Patients with Major Depressive Disorder Using Ultra-High Performance Liquid Chromatography Coupled with Mass Spectrometry
Source: Metabolites. 2021 Jul 20;11(7):466. doi: 10.3390/metabo11070466 (PMC8306682; doi:10.3390/metabo11070466)

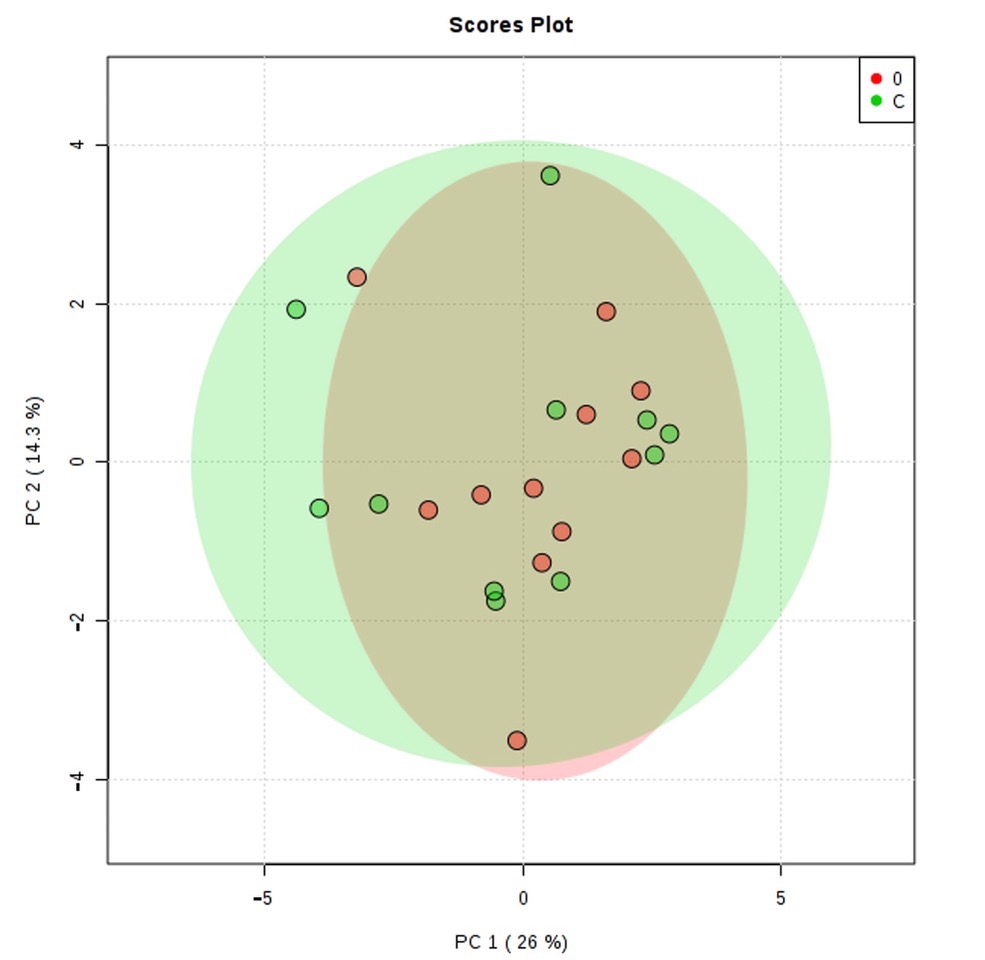

Supplement: Supplementary file 1 [file metabolites-11-00466-s001.zip › Fig S1.jpeg]

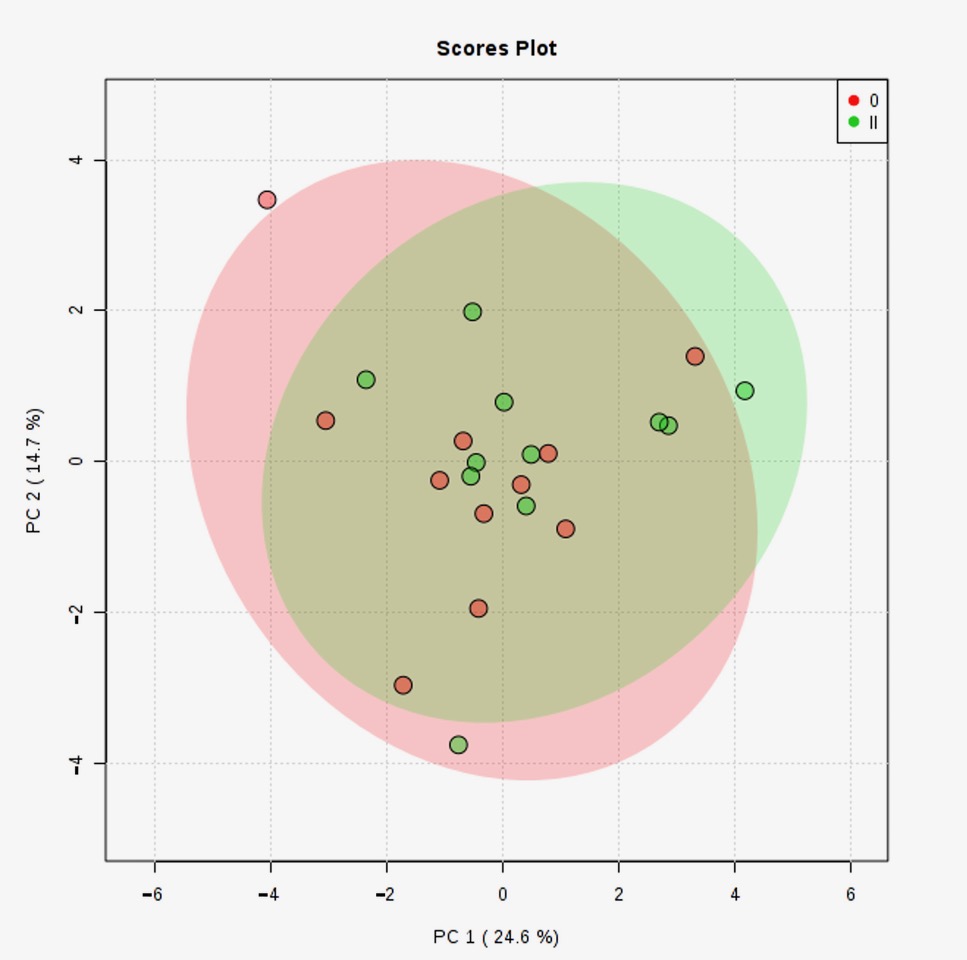

Supplement: Supplementary file 1 [file metabolites-11-00466-s001.zip › Fig S2.jpeg]
